# Effects of Simulated Microgravity on Embryonic Stem Cells

**DOI:** 10.1371/journal.pone.0029214

**Published:** 2011-12-21

**Authors:** Yulan Wang, Lili An, Yuanda Jiang, Haiying Hang

**Affiliations:** 1 National Laboratory of Biomacromolecules, Institute of Biophysics, Chinese Academy of Sciences, Beijing, China; 2 Center for Computational and Systems Biology, Institute of Biophysics, Chinese Academy of Sciences, Beijing, China; 3 Center for Space Science and Applied Research, Chinese Academy of Sciences, Beijing, China; University of California Merced, United States of America

## Abstract

There have been many studies on the biological effects of simulated microgravity (SMG) on differentiated cells or adult stem cells. However, there has been no systematic study on the effects of SMG on embryonic stem (ES) cells. In this study, we investigated various effects (including cell proliferation, cell cycle distribution, cell differentiation, cell adhesion, apoptosis, genomic integrity and DNA damage repair) of SMG on mouse embryonic stem (mES) cells. Mouse ES cells cultured under SMG condition had a significantly reduced total cell number compared with cells cultured under 1 g gravity (1G) condition. However, there was no significant difference in cell cycle distribution between SMG and 1G culture conditions, indicating that cell proliferation was not impaired significantly by SMG and was not a major factor contributing to the total cell number reduction. In contrast, a lower adhesion rate cultured under SMG condition contributed to the lower cell number in SMG. Our results also revealed that SMG alone could not induce DNA damage in mES cells while it could affect the repair of radiation-induced DNA lesions of mES cells. Taken together, mES cells were sensitive to SMG and the major alterations in cellular events were cell number expansion, adhesion rate decrease, increased apoptosis and delayed DNA repair progression, which are distinct from the responses of other types of cells to SMG.

## Introduction

Spaceflight results in a number of adverse effects such as bone loss [1], skeletal muscle atrophy [2], cardiovascular problems [3], immune system dysregulation [4], and alteration of sleep and circadian rhythms [5]. Of all the known space environmental factors, microgravity (MG) has been recognized as a major environmental factor. Many of the above mentioned problems are due to the effects of MG at cellular level. Because of the cost effectiveness and limited access to space flight, simulating MG on Earth is widely used in space life research. Ground-based SMG conditions can be achieved through the use of 3-D clinostats. A 3-D clinostat is a device that generates multidirectional G force and cancels the cumulative gravity, thus effectively simulating certain aspects of MG. Using such devices, detailed experiments have been performed towards the understanding of the effects of MG on many cellular activities.

Studies from ground-based MG simulators have revealed that many cell types, ranging from bacteria to mammalian cells, are sensitive to the MG environment. The major affected cellular activities and parameters are cell proliferation, cell cycle, cell differentiation, apoptosis, genomic integrity and DNA damage repair. Degan et al [6] reported that exposure of human lymphocytes and lymphoblastoid cells to SMG strongly affected energy metabolism and DNA repair. Coinu et al [7] found that SMG induced partial arrest in G2/M phase and increased p14-3-3, HSP70, HSP60 and p21 expression in both normal vascular smooth muscle cells and neoplastic human breast cancer cells (MCF-7). Takeda et al [8] also reported that SMG inhibited cell growth in malignant glioma cells, but they did not observe significant change in any phase of the cell cycle by flow cytometry assay, suggesting that the cell growth inhibition was due to a slowdown of the processions of all the cell cycle phases. Makihira et al [9] reported that long-term exposure (1 d, 3 d, 5 d, 7 d) to SMG causes the decrease in osteoblast differentiation, possibly by suppression of the RANKL-dependent signal pathway and a reduction of RANKL expression in osteoblasts. Thus, the responses of the cells to SMG vary among different cell types.

Compared with many types of differentiated cells and adult stem cells, our knowledge on the cellular responses of mES cells to SMG is rare. Stem cells are undifferentiated cells capable of self-renewal, extensive proliferation and differentiation into one or more cell types. The two broad types of mammalian stem cells are ES cells and adult stem cells. Adult stem cells are crucial for physiological tissue renewal and regeneration after injury. The studies on the responses of adult stem cells such as rat bone marrow mesenchymal stem cells, human mesenchymal stem cells and human hematopoietic progenitor cells to microgravity have been reported by several groups [10], [11], [12], [13]. The results of these studies were to understand the pathogenesis of the abnormalities such as bone loss, anemia and immunodeficiency observed in space. ES cells can be isolated from the inner cell mass of the blastocyst and propagated and maintained in culture. Different from the adult stem cells which are capable of giving rise only to cells of their tissue of origin, ES cells can give rise to nearly all the terminal cell types of the organism [14]. Investigation on the response of ES cells to SMG will help us understand the effect of MG on the reproduction system and embryonic development. Nowadays, ES cells and their differentiated progeny offer tremendous potential for regenerative medicine [15]. In addition, the usage of rotary microgravity clinostats as bioreactors to provide clinically relevant numbers of homogenous therapeutic cell populations for stem-cell-based therapies, especially in the field of ES-derived lineage-specific stem cell induction has aroused much attention [16], [17], [18]. Therefore, it is important for us to understand the basic cell biology of mES cells under SMG.

In this study, using a 3-D clinostat, we systematically studied a set of effects of SMG on mES cells. Mouse ES cells were exposed to SMG for different times to observe time-dependent effects of SMG exposure. We have found that mES cells demonstrate a set of responses to SMG including alterations in cell number expanding capacity, adhesion rate, apoptosis and DNA damage repair, and this set of responses is distinct from those of other types of cells.

## Results

### Effect of SMG on cell differentiation

**Figure 1:**
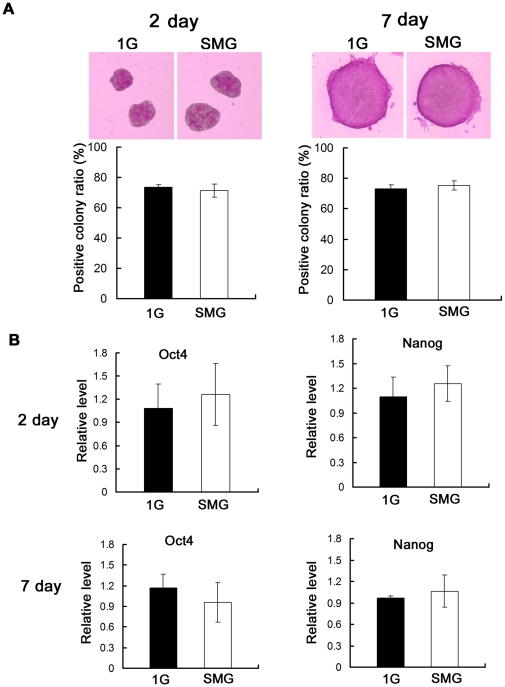
Differentiation state of mES cells after incubation under SMG or 1G. (A) Upper: Representative microphotographs of mES cells of group 1G and group SMG after ALP staining on Day 2 and Day 7, taken at 100× and 200× magnification respectively. Lower: Percentages of ALP-positive colonies on Day 2 and Day 7. More than 50 colonies of each group were checked for the calculation of the positive colony ratio. (B) Evaluation of the relative expression level of Oct4 and Nanog by quantitative real-time PCR on Day 2 and Day 7. Oct4 and Nanog are extensively used pluripotency markers of stem cells. The data represents mean ± SD of three independent experiments.

Differentiation is one of the key features of stem cells, and that the effects of SMG on the differentiation of mES cells have attracted much attention. In this study, we cultured mES cells in the conventional culture condition which maintain mES cells at pluripotent status [19], [20] and investigated the effect of SMG on the differentiation of mES cells. Both the 1G and SMG group cells formed compact colonies with spherical morphology and poorly delineated cell-cell borders, which are characteristic of undifferentiated stem cell morphology, during seven days' conventional culture. Widespread expression of the alkaline phosphatase (ALP) in ES cells makes itself a pluripotent marker [21]. Then we tested the ALP activity of these colonies and calculated the positive colony ratio. As shown in Figure 1A, on day 2 and day 7 of the incubation, cell colonies from the SMG group and the 1G group were both positive for ALP. No significant differences in the ratio of ES cell colonies positive for ALP activity were observed between these two groups. This result indicates that mES cells cultured under SMG could maintain the undifferentiated state. To be more conclusive, we further analyzed the expression of Oct4 and Nanog (widely used pluripotency markers of stem cells) using quantitative real-time PCR. Real-time PCR yielded results similar to those of ALP staining. On day 2 and day 7 of the rotation, there was no significant difference between the SMG group and the 1G group cells in the expression of these two genes (Figure 1B), confirming that the culture under SMG condition did not induce differentiation of mES cells.

### Effect of SMG on cell number expansion

**Figure 2:**
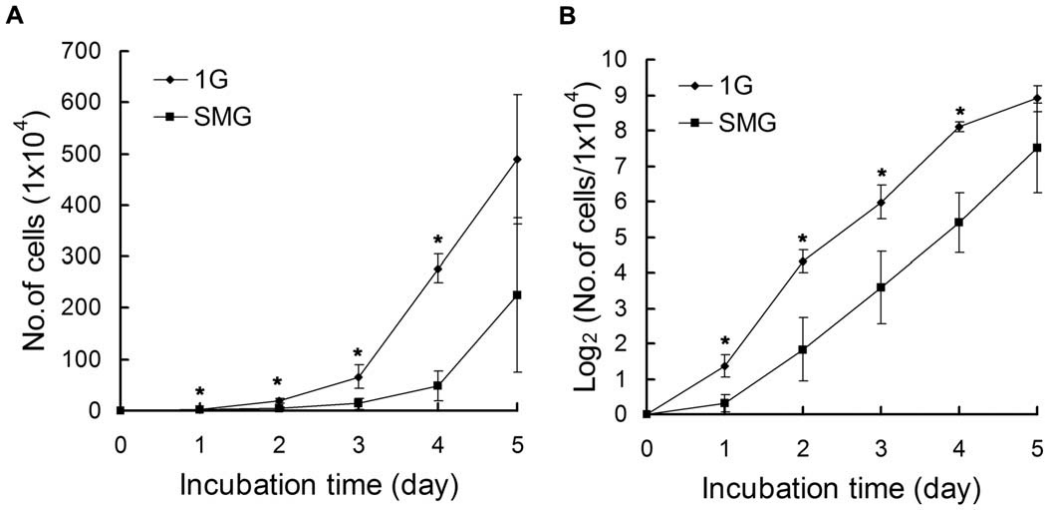
Cell Number Expansion analysis of mES cells after five days incubation under SMG or 1G. The initial cell seeding number was 1×10^4^. (A) Cell number determined by counting cells at indicated time points with a standard hemocytometer. (B) The doubling generation curve generated by dividing the cell number with 10^4^ and then transferring the quotient to the logarithm to the base 2. The data represents mean ± SD of three independent experiments. Student's t test, *p<0.05.

Cell number expansion was reported to be affected by SMG in many types of cells. Here we analyzed the expansion of the 1G and the SMG group cells by counting the cells on day 1, 2, 3, 4 and 5. As shown in Figure 2A, there were significantly fewer cells in the SMG group compared to the 1G group (1.2×10^4^ versus 2.6×10^4^ on day 1, p = 0.04; 4×10^4^ versus 20×10^4^ on day 2, p = 0.01; 14.2×10^4^ versus 65.7×10^4^ on day 3, p = 0.04; 47.9×10^4^ versus 277.2×10^4^ on day 4, p = 0.00061 and 225.3×10^4^ versus 488.3×10^4^ on day 5, p = 0.08). In order to further investigate the cell proliferation dynamics, we also used the doubling generation curve. For simplicity, the original cell number was divided by 10^4^. Then the quotient was transferred to the logarithm to the base 2, and the data were replotted into a semi-log curve (also called doubling generation curve) which can reflect cell proliferation more accurately than a linear curve. As shown in Figure 2B, the doubling generation curve of the 1G group went straight up with a slight slant at day 1 and day 5, while the doubling generation curve of the SMG group encountered a big turn at day 1 and then went straightly up. Interestingly, compared to the 1G group, the slope of the doubling generation curve of the SMG group was lower on day 1 and day 2. From day 3 to day 4, the slopes of the doubling generation curves of the two groups were almost identical. This data suggest that the major effects of SMG on cell expansion were acutely sensed in the first two days and counterbalanced afterwards by mES cells.

**Figure 3:**
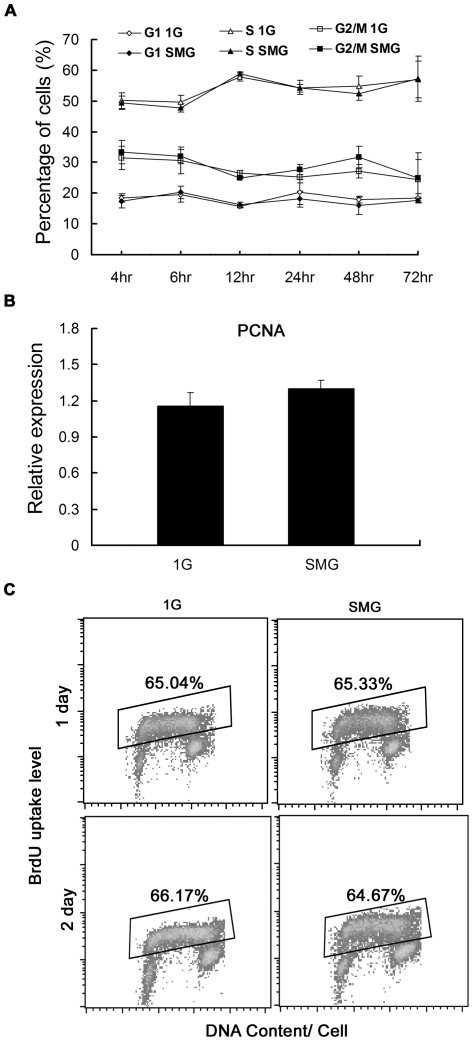
Cell proliferation analyzed by three independent methods from three different aspects. Mouse ES cells were cultured under SMG or 1G for indicated time points. Then cells were processed properly for the following analysis: (A) Quantitative comparison of cell cycle distribution between the 1G group and SMG group. Cell cycle distribution was determined by flow cytometry at 4 hr, 6 hr, 12 hr, 24 hr, 48 hr and 72 hr. The data represents mean ± SD of three independent experiments. (B) Evaluation of relative expression levels of PCNA by quantitative real-time PCR on Day 2. The data represents mean ± SD of three independent experiments. (C) Flow cytometry analysis of cells stained with both PI and BrdU after 1day and 2days incubation. BrdU-positive S phase cells were gated. Experiments were performed thrice and representative analyzes are shown.

The impaired expansion might be caused by impaired cell proliferation, decreased attachment ability of the cells to the flask wall and/or increased apoptosis. We analyzed the cell proliferation by investigating the cell cycle distribution, PCNA expression and BrdU-uptake. We checked the cell cycle distribution using flow cytometry to investigate whether SMG could induce cell cycle arrest in mES cells. The time points were 4 hr, 6 hr, 12 hr, 24 hr, 48 hr and 72 hr after the cells were cultured under SMG. As shown in Figure 3A, the cell cycle distribution among different time points consisted of small fluctuations. Furthermore, about half of the cells in both groups were in S phase and this was consistent with the well known characteristics of ES cells that they are capable of extensive proliferation. Interestingly, although we observed lower expansion rates of SMG group cells on day 1 and day 2, there were no significant differences in the cell cycle distribution especially the percentage of S phase cells between the 1G group cells and the SMG group cells on each time point detected. PCNA is a nuclear protein that is differentially expressed during the cell cycle with the peak in S phase and has been broadly used as a marker of active cell proliferation. We detected PCNA expression on day 2 using quantitative real-time PCR and consistent with the results of cell cycle distribution mentioned above, there was no significant difference in PCNA expression between the 1G group cells and the SMG group cells as shown in Figure 3B. BrdU can be incorporated into newly synthesized DNA strands of actively proliferating cells, thus allowing the assessment of the population of cells which are actively synthesizing DNA. Compared with the 1G group, there was no significant difference in the number of BrdU-positive S phase cells in the SMG and 1G groups (Figure 3C) and so was the intensity of BrdU uptake. In summary, our results indicate that SMG did not affect the proliferation of mES cells.

**Figure 4:**
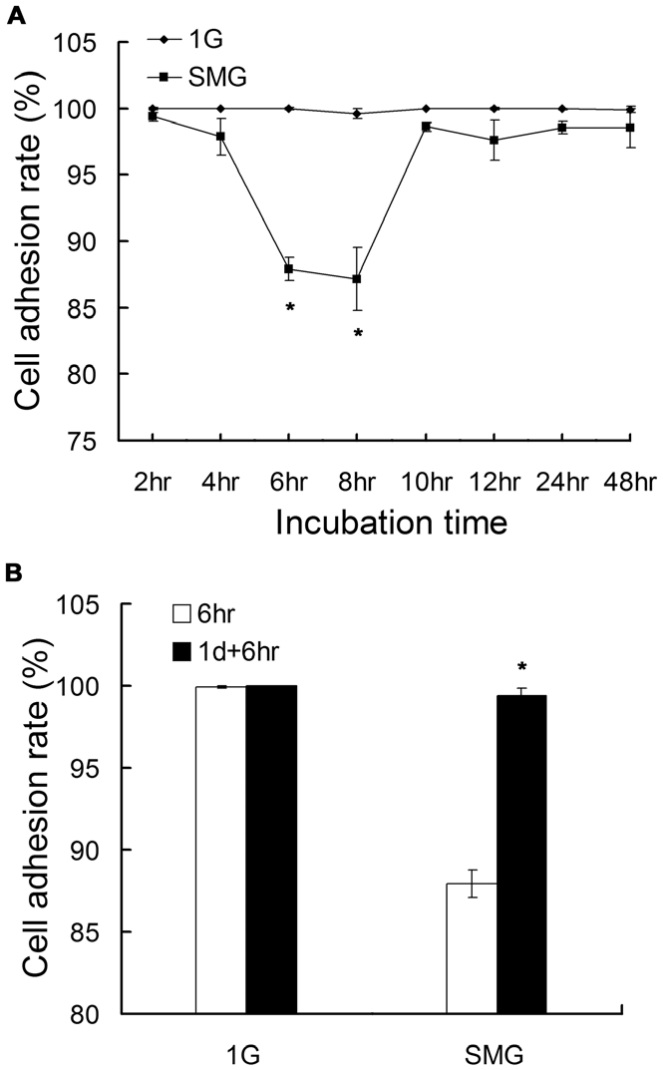
Microgravity induced decrease in adherent mES cells at early exposure hours. Mouse ES cells were cultured in conventional culture condition under SMG or 1G. At indicated time points, adherent cells and detached cells were collected separately. The adhesion rate is the ratio of adhesive cells to the sum of adherent cells and detached cells. (A) The adhesion rate at 2 hr, 4 hr, 6 hr, 8 hr, 10 hr, 12 hr, 24 hr and 48 hr. (B) Prolonged exposure time to MG and the enhanced adhesion rate. Cells in Group 1 d+6 hr were cultured for 1 day and then the detached and dead cells were discarded by replacing the culture media with new ones. After that, cells were incubated for another 6 hours. The data represents mean ± SD of three independent experiments. Student's t test, *p<0.05.

We then studied whether SMG could affect the attachment ability of the cells to the flask wall. As shown in Figure 4A, compared to the 1G group, the adhesion rates in the SMG group were reduced to the lowest levels at 6 hr and 8 hr of culture. At other time points, only very slight reduced adhesion rates were observed in the SMG group. We speculate that cells made quick adjustments to enhance their adhere abilities. To further verify this, we performed the following experiment. After 1day's culture (and after which point the adhesion state was stable), we replaced the culture media with fresh ones in order to exclude the detached cells produced in the first day culture; and then cultured the cells for another 6 hours. Cells were processed as described in materials and methods. The incubation time of these cells was denominated as 1 d+6 hr so as to distinguish itself from the first 6 hr time point. As shown in Figure 4B, in Group SMG, the adhesion rate at 6 hr is 87.9% while at 1 d+6 hr, the adhesion rate is 99.4%. The differences between the two time points were significant (p = 0.002). However no differences were observed in Group 1G (the adhesion rate was 99.93% at 6 hr and 99.97% at 1 d+6 hr respectively). Taken together, the adhesion rate of mES cells under SMG started to decrease between 6 hr and 8 hr of rotation, bounced back to normal level and became stable from 10 hr of rotation.

**Figure 5:**
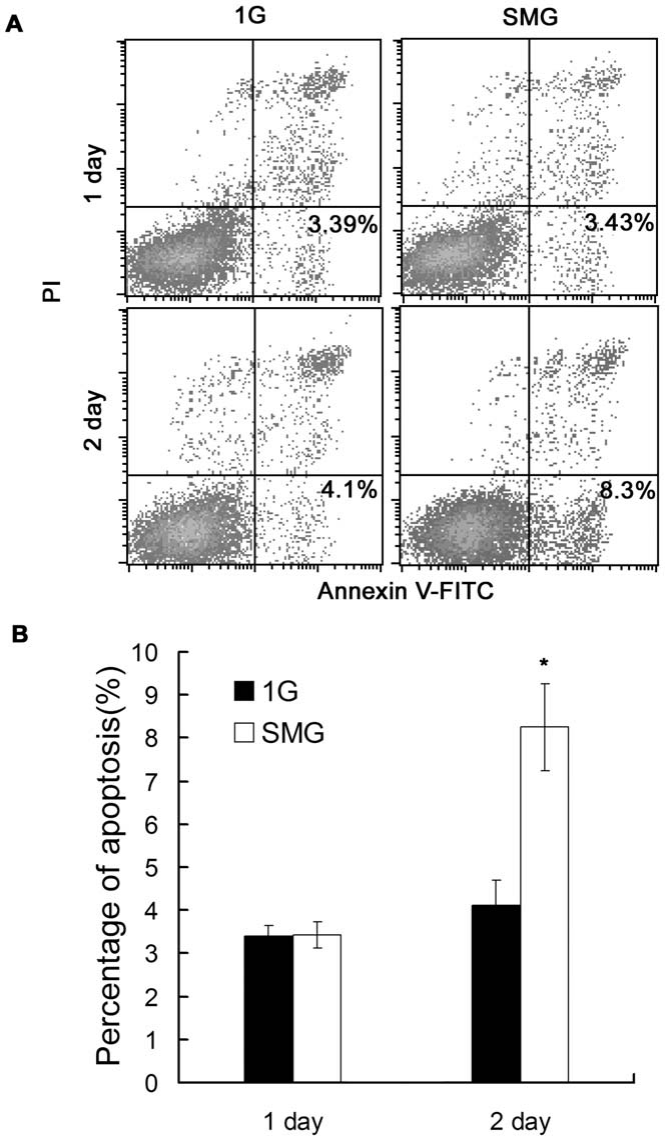
Cell apoptosis analysis of mES cells cultured under SMG or 1G. (A) Flow cytometric analysis of cells to assess apoptosis using Annexin V labeling. Experiments were performed thrice and representative analyzes are shown. (B) Quantitative comparison of apoptosis between the 1G Group and the SMG Group. Three apoptotic assays shown in (A) were performed for comparison. Student's t test, *p<0.05.

We also studied whether SMG could influence the apoptosis of mES cells. Figure 5A shows the representative results of apoptosis analyzed by flow cytometry and figure 5B shows the quantitative comparison results. On day 1, there was no significant difference in the percentage of apoptotic cells (3.43% versus 3.39%, p = 0.8) between the SMG group and 1G group cells. On day 2, the percentages of apoptotic cells were significantly higher in the SMG Group than those in the 1G group (8.3% versus 4.1%, p = 0.007). The result indicates that induction of apoptosis in mES cells under SMG was apparent on the second day of rotation.

Our results indicates that SMG did not affect the proliferation of mES cells and the SMG-induced apoptosis and cell detachment from the flask wall were important factors affecting cell expansion.

### Effects of SMG on DNA damage and DNA damage repair

**Figure 6:**
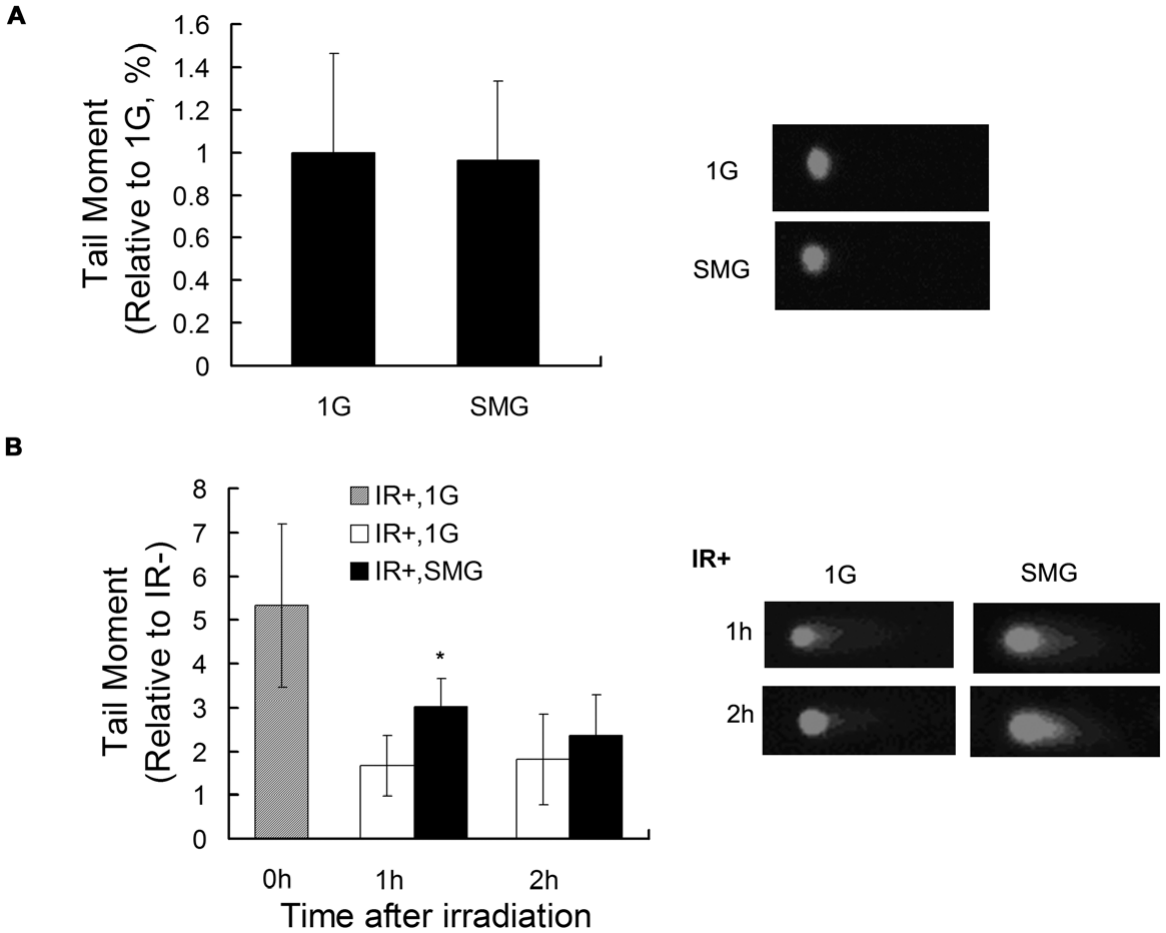
Comet assay analysis of DNA damage and DNA damage repair under SMG or 1G. (A) Evaluation of DNA damage by the alkaline comet assay in mES cells after two days incubation under SMG or 1G. (B) Kinetics of comet tail moment in mES cells irradiated with 8 Gy of gamma-ray and incubated in 1G or in SMG evaluated by neutral comet assay. Time points were 0 hr, 1 hr, 2 hr. On the right representative comet assay results were shown, and on the left quantitative comparison of comet tail moments between the 1G group and the SMG group. At least 50 cells of each time point was scored for comet tail moment. The data represents mean ± SD of three independent experiments. Student's t test, *p<0.05.

The alkaline comet assay is a sensitive method for measuring DNA lesions (including single and double strand breaks and base modifications) and detecting repair kinetics in single cells. The amount of DNA migration under electric potential indicates the amount of DNA damage in the cell. In the above experiments, we observed that the major biological changes of clinorotated mES cells happened within 2 days of culture. Therefore, we first measured the effect of SMG on genomic integrity of mES cells after 2 days' treatment using the alkaline comet assay. As shown in Figure 6A, no apparent tails were detected in both the 1G group and the SMG group cells on day 2. These data suggest that SMG alone does not induce DNA lesions in mES cells.

Next, we investigated whether SMG could affect the repair of radiation-induced DNA lesions of mES cells. Mouse ES cells exposed to 8Gy gamma-ray radiation (IR+) or not exposed (IR-) were incubated for 0 hr, 1 hr and 2 hr respectively in the 1G or SMG condition. Neutral comet assay was performed to measure DNA double strand breaks (DSB) induced by ionizing radiation. The progression of DSB repair was indicated by the loss of the comet tail. On the left of Figure 6B, a statistical result of the comet tail moment was shown. At 0 hr after radiation, as expected, the tail moment was significantly longer than the other time points, indicating that the DNA damage induced by gamma-ray radiation was gradually repaired as time went by. However the progression of repair was different under different gravity conditions. At 1 hr after radiation, the tail moment of cells cultured in SMG was significantly longer than that of cells in 1G. At 2 hr, there was also difference in tail moment between the two groups of cells though not significant at this experimental setting. This indicates that SMG can impair the early-stage repair of radiation-induced DNA lesions of mES cells.

## Discussion

In this study, we have systematically investigated the effects of SMG on mES cells and we found that mES cells were sensitive to SMG and the major alterations in cellular events were population growth inhibition, decreased adhesion rate, increased apoptosis and delayed DNA repair progression, which are unique to the responses of other types of cells to SMG.

We first detected the effect of SMG on the cell number expansion of mES cells and observed that the cell number was significantly reduced in the SMG group than that in the 1G group (Fig. 2A). It seems that SMG impaired the expansion of mES cells throughout the whole process of the 5 days' treatment. However, after we transformed the data to the doubling generation curve and calculated the doubling time, we found that the doubling time of the SMG group was longer than that of the 1G group only from day 0 to day 2 (26.1 hr versus 11.1 hr), and from day 3 to day 4, the doubling time of the two groups were almost identical (13.4 hr versus 12.6 hr), indicating an adaptation of cells to SMG. From day 4 to day 5, the slope of the doubling generation curve of the 1G group was smaller than that of the SMG group because of the reaching of the plateau phase (data not shown here). These data suggest that major effects of SMG on cell expansion were acutely sensed in the first two days and counterbalanced afterwards by mES cells. There were also reports about the effects of SMG on cell expansion in other cell types. For example, in normal vascular smooth muscle cells, human breast cancer cells [7], malignant glioma cells [8] and human mesenchymal stem cells [13] cell expansion was affected, while the effect on cell expansion was not observed in normal human osteoblasts [22]. Thus the effects of SMG on cell expansion vary among different cell types. However, they did not characterize the cell doubling time as we did in this study, so detailed population kinetic information such as proliferation adjustment to SMG in these studies was unknown.

The impaired cell number expansion might be caused by impaired cell proliferation, decreased attachment ability of the cells to the flask wall and/or increased apoptosis. We observed that no specific cell cycle phase was significantly altered under SMG conditions, suggesting that there was no significant difference in cell proliferation between the 1G and SMG groups. This was further verified by the expression of PCNA, a broadly used marker of cell proliferation [23], [24], [25]. Interestingly a similar result was reported by Takeda et al [8]. They found that cell expansion in malignant glioma cells was inhibited under SMG without evident change in any phase of the cell cycle. However, mitochondrial activity did decrease under SMG. They argued that, under SMG conditions, cells enter a hibernation-like state, without any cell cycle arrest but a slowdown of the whole cell cycle. Another way to check whether there is a cell hibernation-like state was to analyze the BrdU uptake. In principle, slowed cell cycle speed coupled with slowed BrdU incorporation during the limited time of pulse-labeling, resulting in less BrdU uptake. In our study, however, the BrdU uptake was not less in the SMG group than in the 1G group, suggesting the cell cycle progress of mES cells was not slowed down, thus no cell hibernation occurred. However, we observed increased apoptosis and decreased adhesion rate of mouse ES cells under SMG. Taken together, our results indicates that SMG did not affect the proliferation of mES cells and the SMG-induced apoptosis and cell detachment from the flask wall were important factors affecting cell expansion.

Interestingly, we found that mES cells started to detach from the flask between 6 hr and 8 hr of rotation in SMG treatment, and the adhering ability bounced back to normal level quickly at 10 hr of SMG treatment, indicating that cells made quick adjustments to enhance their adhering abilities. As discussed in the above paragraph, cell expansion rate also demonstrates adaptation behavior to SMG. It is likely that many more cell activities adjust to MG. Sarkar et al [26] cultured the osteoblast-like ROS 17/2.8 cells under SMG condition, and observed that there were significantly more detached cells in the SMG group than that in the control groups at 24 hr and the cells became confluent in both the SMG group and the control groups at 72 hr. Consistent with our findings, this phenomenon might be due to adaptation of osteoblasts to SMG. However, it seems that the adaptation time needed for mES cells was much shorter than osteoblasts. As to what underlying molecular adjustments have been made need further investigation.

Gelatin-coated or feeder cell-coated plates/flasks are commonly used for culturing mES cells. In this study, we cultured mES cells on feeder cell-free and gelatin-coated flasks. On gelatin-coated flasks, mES cells demonstrated a brief period of detachment from the flask wall and apoptosis. We wondered if these effects were depended on the coating material. The information on other coating conditions than gelatin in the culture of mES cells is rare. Hayashi et al [27] compared the effects of different coating conditions on mES cells in a defined serum-free culture medium. They tested seven extracellular matrix (ECM) components for their effects on mES cell differentiation, and they found that poly-D-lysine (PDL) could also maintain the undifferentiated state of mES cells efficiently as gelatin among the ECM components they tested. Thus we chose PDL as another coating condition and evaluated the cell attachment and apoptosis under PDL coating. Compared to the 1G group, the adhesion rate of the SMG group cells was lowest at 6 hr and 8 hr. After that, the adhesion rate bounced back, and this trend of change is similar to what we observed in the cells cultured in gelatin-coated flasks although the overall adhesion rates of the cells cultured in PDL-coated flasks in the SMG group were lower (Figure S1). However, we did not observed a higher apoptosis level in cells in the SMG group than that in the 1G group in PDL-coated flasks, in contrast to the phenomenon in gelatin-coated flasks (Figure S2). Therefore, the apoptosis increase is a coating-material dependent phenomenon. Even without the apoptosis increase, mES cells also demonstrated a cell number expansion reduction in the SMG group in PDL-coated flasks similar to that in gelatin-coated flasks (Figure S3). It is likely that the overall lower attachment rate and a short period of detachment play a major role in cell number expansion reduction of mES in PDL-coated flasks under SMG condition.

The effects of SMG on genomic stability have been investigated by several groups and SMG was found to induce genomic DNA damage in lymphocytes with extended period of exposure (7 days) to SMG [28], but not in human lymphocytes and lymphoblastoid cells exposed to SMG for 8 hr or 24 hr [6]. In this study, we did not observe increased DNA lesions in mES cells after two days' exposure to SMG, and our result is in consistent with the findings reported by Degan et al [6], indicating that SMG alone could not induce DNA damage in mES cells after short time of exposure.

Other than SMG, radiation is another important environmental factor during spaceflight, which could cause severe DNA lesions. In the present study, we also investigated the repair of radiation-induced DNA lesions in mES cells under SMG. We found that SMG impaired the repair capacity of mES cells at 1 hr post-irradiation while at 2 hr post-irradiation, there was no significant difference between the 1G and SMG group. Therefore, we speculate that SMG impairs the repair of radiation-induced DNA lesions only at the early stage of the lesion repair. Mognato et al also reported that in human peripheral blood lymphocytes (PBLs), the mutant frequency induced by IR was increased by incubation in SMG [29] and the SMG incubation during DNA repair delayed the rate of radiation-induced DSB rejoining [30]. However, in their reports the effects could be observed 24 hr after IR and SMG treatment and they did not show the data of more than 24 hr, obviously different from the short effects of SMG on the DNA repair capacity of mES cells observed in this study.

We observed that the attachment of mES decreased and then bounced back after exposure to SMG. Hammond et al [31]compared the expression of 10000 genes of primary cultures of human renal cortical cells grown under the culture condition including MG, rotating wall vessel, 3-g centrifuge and static culture. They found that the genes whose expression changed the most include adhesion molecules, cytoskeletal protein genes and apoptosis genes. Furthermore, cytoskeleton abnormalities have been observed in the studies conducted on different types of cells such as osteoblastic ROS 17/2.8 cells [32], glial cells [33], human MCF-7 cells [34]. Under SMG, the cytoskeleton of these cells was disorganized at the beginning and after a certain time was reestablished. Interestingly, this accords with our findings of the adaptation of mES cells to SMG (proliferation rate and detachment). Interestingly, cytoskeleton is known to be crucial for numerous cellular processes, including cell adhesion. All the above studies used differentiated cells. Here we showed that stem cells had the bounce-back phenomenon in adhesion to the flask, thus the mechanisms behind the adhesion bounce-back may involve the induction of adhesion and cytoskeleton genes and warrant further investigation.

In this study, we investigated the unique responses of mES cells to SMG. We found that mES cells were sensitive to SMG at the early stage of SMG treatment and the cells could make quick adjustments to adapt to the SMG condition (proliferation rate and detachment). Our results provide new information on the effects of SMG on mES cells. Such information is novel in ES cell biology and may be helpful in stem cell based regenerative medicine.

## Materials and Methods

### 3D-clinostat

The 3D-clinostat, a multidirectional G force generator, was produced by Center for Space Science and Applied Research, Chinese Academy of Sciences [35]. By employing a simultaneous rotation on two axes, the 3D-clinostat is able to produce an environment with an average of 10^−3^ G, thus simulating MG conditions.

### ALP staining

The alkaline phosphatase activity of mES Cells was detected using an Alkaline Phosphatase Detection Kit (Millipore Co.) according to the manufacturer's protocol. The stained cells were examined using an inverted phase-contrast microscope. A colony in which all the cells were positively stained with Fast Red Violet was defined as an undifferentiated colony. We characterized more than 50 colonies of each sample to generate a positive colony ratio.

### Mouse ES cells culture

Mouse ES cells, originally derived from Joyner's laboratory [36], [37], were maintained on gelatin-coated flasks in standard ES cell medium in the presence of leukemia inhibitory factor (LIF) according to Joyner AL without a feeder layer [38]. Cells were seeded in culture flasks (Becton Dickinson) and were cultured under a 1G environment for 18 hr to achieve adhesion. Then the flasks were filled with fresh complete medium to eliminate air bubbles and to diminish turbulence and shear forces. The samples were randomized to two groups. One group was cultured in the 3D-clinostat (group SMG) and the other group was cultured in a normal 1G environment (group 1G). The system was placed in an atmosphere of 95% air/5% CO_2_ at 37°C. The day on which the cells were mounted on the clinostat was referred to as Day 0. The culture medium was not changed during the experimental period.

### Cell number expansion analysis

Mouse ES cells were seeded at a concentration of 1×10^4^ cells per 25 cm^2^ culture flask and the samples were handled as mentioned above. At the indicated time points (day 1, day 2, day 3, day 4 and day 5) the attached cells were collected by trypsinization and the cell numbers were determined using a hemocytometer.

### Cell adhesion assay

Mouse ES cells were seeded at a concentration of 1×10^5^ cells per 12.5 cm^2^ culture flask and the samples were handled as mentioned above. At the indicated time points (2 hr, 4 hr, 6 hr, 8 hr, 10 hr, 12 hr, 24 hr and 48 hr), the cells adhere to the flask or the cells in the medium were collected separately and the cell number was estimated using a hemocytometer. The adhesion rate is the ratio of adhesive cells to the sum of adhesive cells and detached cells in the medium.

### Cell cycle analysis

The profile of cells in different phases of the cell cycle was determined using previously established methods [39]. Mouse ES cells were seeded at a concentration of 5×10^5^ cells per 25 cm^2^ culture flask. After incubation for indicated times (4 hr, 6 hr, 12 hr, 24 hr, 48 hr and 72 hr), cells were processed and stained with propidium iodide (PI), then analyzed by a FACSCalibur (Becton Dickinson).

### Apoptosis assays

Mouse ES cells were seeded at a concentration of 5×10^5^ cells per 25 cm^2^ culture flask. Cultured cells were trypsinized for 3 min using 0.1% trypsin at 37°C (Sigma), washed twice with cold PBS, then resuspended in 1× binding buffer [10 mmol/L HEPES (pH 7.4), 140 mmol/L NaCl, and 2.5 mmol/L CaCl_2_] at a concentration of 1×10^6^ cells per milliliter. Then cells were stained with Alexa Fluor® 488 annexin V and PI (Invitrogen) for 15 min at room temperature, before flow cytometric analysis.

### BrdU uptake assays

Mouse ES cells were seeded at a concentration of 5×10^5^ cells per 25 cm^2^ culture flask and the cells were cultured under 1G and SMG conditions for the indicated times (1 day and 2 days). Culture medium was replaced with fresh ones containing 20 µM BrdU and cells were pulse-labeled for 10 min. Cells were then processed and probed with FITC-conjugated anti-BrdU antibody (Becton Dickinson), and stained with PI. Flow cytometric analyzes were performed on a FACSCalibur (Becton Dickinson).

### Comet assay

The protocol published by Singh et al [40] was used with minor modifications. The slides were pre-coated with a thin layer of 1% normal melting agarose and allowed to dry. Single cell suspensions of either SMG-treated or control cells were harvested and re-suspended to 5×10^5^ cells/ml. 20 µl of each final suspension was added to 80 µl of pre-melted 0.75% low melting agarose and was pipetted onto the pre-coated slide. After solidification, the slides were placed in neutral/alkaline lysis solution and the cells were lysed in the dark at 4°C for 2 hour. Slides were then placed in 1×TBE/alkaline buffer in the dark at 4°C for 20 minutes to allow for unwinding of the DNA. The slides were subjected to electrophoresis at ∼0.74 V/cm for 20 min. Following electrophoresis, the slides were stained with PI. Fluorescence images were captured using a microscope and analyzed by CASP-1.2.2 software (University of Wroclaw) for tail moment (the geometric mean of fluorescence on the tail from the nucleus) [41].

### Quantitative real-time PCR analysis

Total RNA was prepared from cultured cells using the RNeasy Mini kit (QIAGEN), as described by the manufacturer. For reverse transcription-PCR (RT-PCR), 2 µg total RNA were reverse transcribed in a 20 µL reaction volume to form cDNA using the SuperScript First-Strand Synthesis System for RT-PCR (Invitrogen). Real-time PCR was performed using the LightCycler system with FastStart DNA Master SYBR Green I to label amplified DNA (Roche). A standard curve method of quantification was used to calculate the expression of target genes relative to the housekeeping gene GAPDH. Experiments were performed thrice. The following primer pairs were used for the PCR reactions: PCNA, 5′-TTTGCACGTATATGCCGAGAC-3′ and 5′-GGTGAACAGGCTCATTCATCTCT-3′; Oct4, 5′-AGTTGGCGTGGAGACTTTGC-3′ and 5′-CAGGGCTTTCATGTCCTGG-3′; Nanog, 5′-TTGCTTACAAGGGTCTGCTACT-3′ and 5′-ACTGGTAGAAGAATCAGGGCT-3′; GAPDH, 5′-TGAAGCAGGCATCTGAGGG-3′ and 5′-CGAAGGTGGAAGAGTGGGAG-3′. PCR procedures for these genes were template denaturation at 94°C for 1 min, then 40 cycles of 94°C for 30 s, 60°C for 30 s, 72.0°C for 20 s, and a final extension at 72°C for 3 min.

### Statistical analysis

The Student's t test was performed to determine statistical significance of the differences. A P value of <0.05 was considered statistically significant.

## Supporting Information

Figure S1 **Microgravity induced decrease in adherent mES cells at early exposure hours.** Mouse ES cells were cultured in conventional culture condition in PDL-coated flasks under SMG or 1G. At indicated time points, adherent cells and detached cells were collected separately. The adhesion rate is the ratio of adhesive cells to the sum of adherent cells and detached cells. The data represents mean ± SD of three independent experiments. Student's t test, *p<0.05.
(TIF)

Figure S2 **Cell apoptosis analysis of mES cells cultured under SMG or 1G.** Mouse ES cells were cultured in conventional culture condition in PDL-coated flasks under SMG or 1G. (A) Flow cytometric analysis of cells to assess apoptosis using Annexin V labeling. Experiments were performed thrice and representative analysis is shown. (B) Quantitative comparison of apoptosis between the 1G Group and the SMG Group. Three apoptotic assays shown in (A) were performed for comparison.
(TIF)

Figure S3 **Cell Number Expansion of mES cells during five days' incubation under SMG or 1G in PDL-coated flasks.** A set of mES cells of 1×10^4^ were seeded in PDL-coated flasks and incubated for 18 hr under a 1G environment to achieve adhesion and then incubated under 1G or SMG conditions for designated times, and cell numbers were counted. (A) Cell number expansion described by linear growth curves. (B) Cell number expansion described by semi-log growth curves. The doubling generation curves (semi-log growth curves) were generated by dividing the cell number with 10^4^ and then transferring the quotient to the logarithm to the base 2. The data represents mean ± SD of three independent experiments. Student's t test, *p<0.05.
(TIF)
