# Supplementary figures and images for: Effects of Simulated Microgravity on Embryonic Stem Cells
Source: PLoS One. 2011 Dec 21;6(12):e29214. doi: 10.1371/journal.pone.0029214 (PMC3244445; doi:10.1371/journal.pone.0029214)

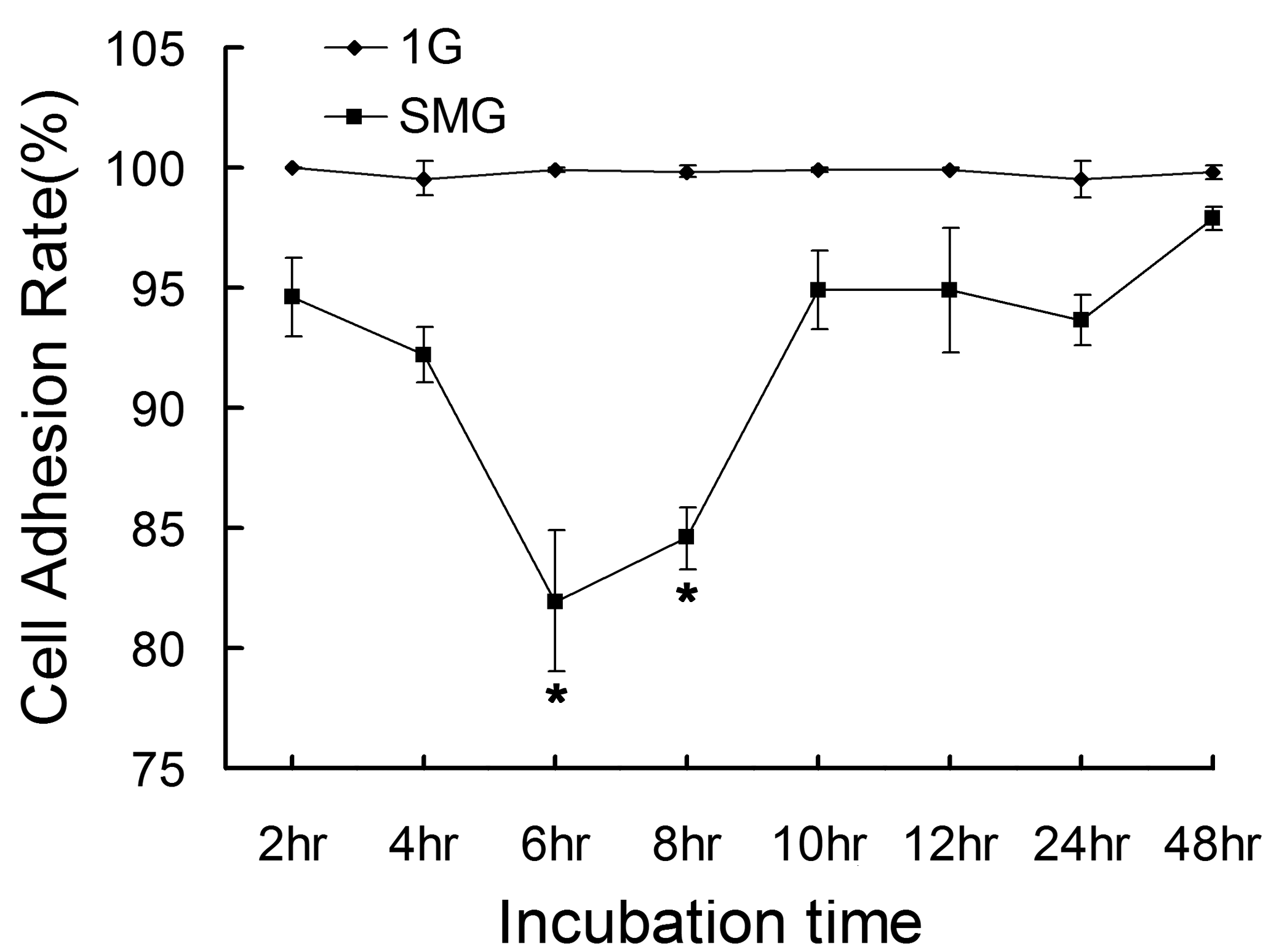

Supplement: Figure S1 — Microgravity induced decrease in adherent mES cells at early exposure hours. Mouse ES cells were cultured in conventional culture condition in PDL-coated flasks under SMG or 1G. At indicated time points, adherent cells and detached cells were collected separately. The adhesion rate is the ratio of adhesive cells to the sum of adherent cells and detached cells. The data represents mean ± SD of three independent experiments. Student's t test, *p<0.05. (TIF) [file pone.0029214.s001.tif]

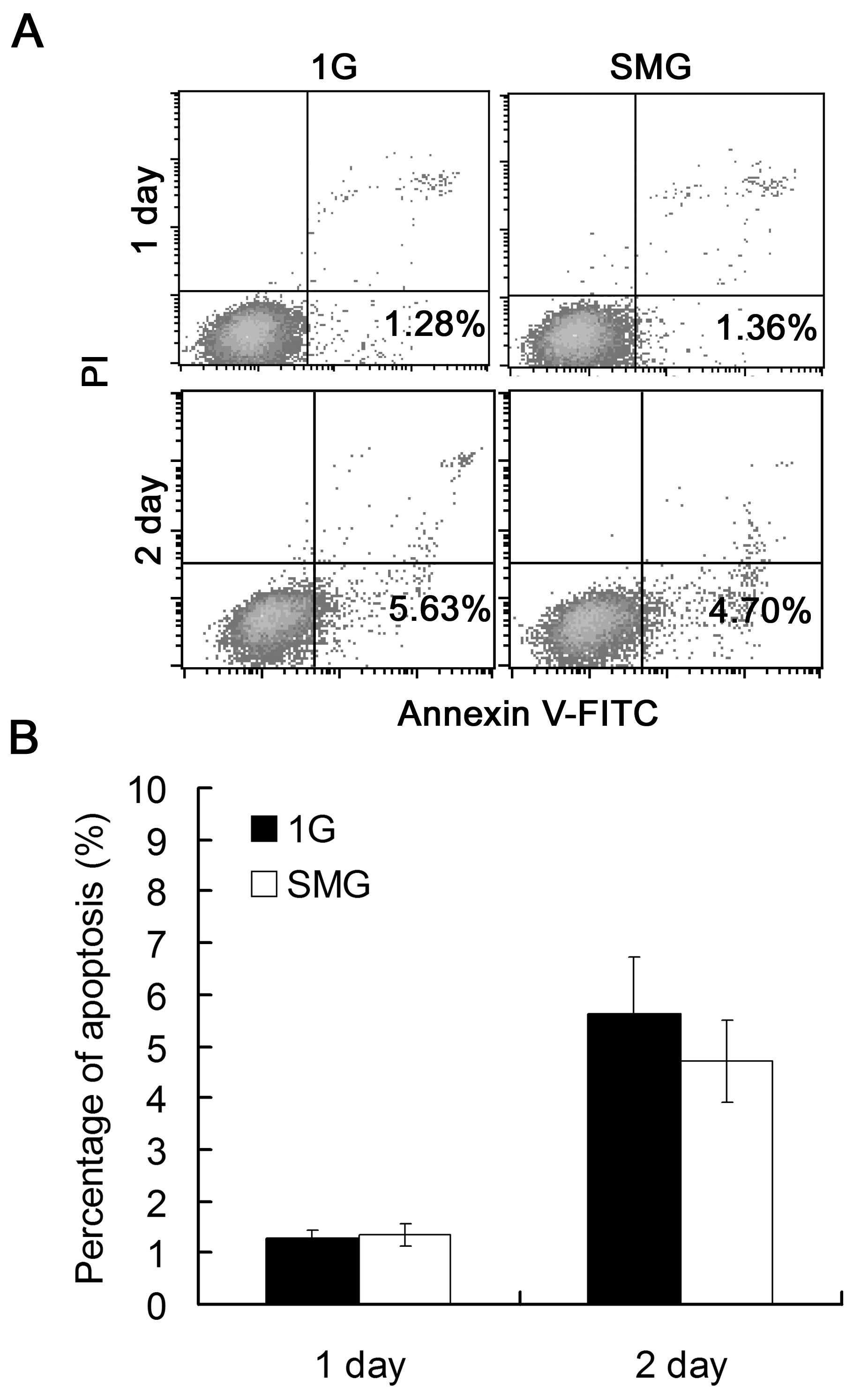

Supplement: Figure S2 — Cell apoptosis analysis of mES cells cultured under SMG or 1G. Mouse ES cells were cultured in conventional culture condition in PDL-coated flasks under SMG or 1G. (A) Flow cytometric analysis of cells to assess apoptosis using Annexin V labeling. Experiments were performed thrice and representative analysis is shown. (B) Quantitative comparison of apoptosis between the 1G Group and the SMG Group. Three apoptotic assays shown in (A) were performed for comparison. (TIF) [file pone.0029214.s002.tif]

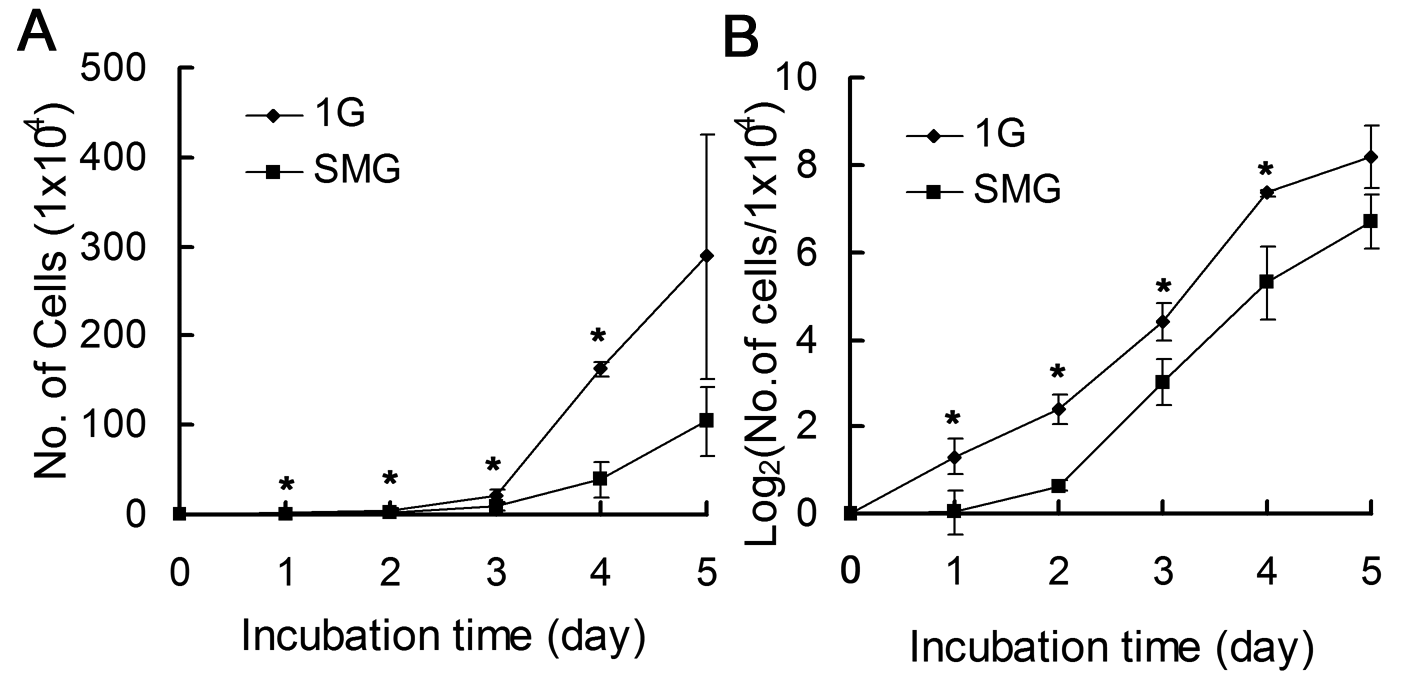

Supplement: Figure S3 — Cell Number Expansion of mES cells during five days' incubation under SMG or 1G in PDL-coated flasks. A set of mES cells of 1×10^4^ were seeded in PDL-coated flasks and incubated for 18 hr under a 1G environment to achieve adhesion and then incubated under 1G or SMG conditions for designated times, and cell numbers were counted. (A) Cell number expansion described by linear growth curves. (B) Cell number expansion described by semi-log growth curves. The doubling generation curves (semi-log growth curves) were generated by dividing the cell number with 104 and then transferring the quotient to the logarithm to the base 2. The data represents mean ± SD of three independent experiments. Student's t test, *p<0.05. (TIF) [file pone.0029214.s003.tif]
